# Identification of potential mediators of the relationship between body mass index and colorectal cancer: a Mendelian randomization analysis

**DOI:** 10.1093/ije/dyae067

**Published:** 2024-05-09

**Authors:** Emmanouil Bouras, Dipender Gill, Verena Zuber, Neil Murphy, Niki Dimou, Krasimira Aleksandrova, Sarah J Lewis, Richard M Martin, James Yarmolinsky, Demetrius Albanes, Hermann Brenner, Sergi Castellví-Bel, Andrew T Chan, Iona Cheng, Stephen Gruber, Bethany Van Guelpen, Christopher I Li, Loic Le Marchand, Polly A Newcomb, Shuji Ogino, Andrew Pellatt, Stephanie L Schmit, Alicja Wolk, Anna H Wu, Ulrike Peters, Marc J Gunter, Konstantinos K Tsilidis

**Affiliations:** Department of Hygiene and Epidemiology, University of Ioannina School of Medicine, Ioannina, Greece; Chief Scientific Advisor Office, Research and Early Development, Novo Nordisk, Copenhagen, Denmark; Department of Epidemiology and Biostatistics, Imperial College London, School of Public Health, London, UK; Department of Epidemiology and Biostatistics, Imperial College London, School of Public Health, London, UK; Nutrition and Metabolism Branch, International Agency for Research on Cancer, Lyon, France; Nutrition and Metabolism Branch, International Agency for Research on Cancer, Lyon, France; Faculty of Human and Health Sciences, University of Bremen, Bremen, Germany; Department Epidemiological Methods and Etiological Research, Leibniz Institute for Prevention Research and Epidemiology, Bremen, Germany; Department of Population Health Sciences, Bristol Medical School, University of Bristol, Bristol, UK; MRC Integrative Epidemiology Unit, University of Bristol, Bristol, UK; Department of Population Health Sciences, Bristol Medical School, University of Bristol, Bristol, UK; MRC Integrative Epidemiology Unit, University of Bristol, Bristol, UK; NIHR Bristol Biomedical Research Centre, University Hospitals Bristol and Weston NHS Foundation Trust and the University of Bristol; Department of Population Health Sciences, Bristol Medical School, University of Bristol, Bristol, UK; MRC Integrative Epidemiology Unit, University of Bristol, Bristol, UK; Division of Cancer Epidemiology and Genetics, National Cancer Institute, National Institutes of Health, Bethesda, MD, USA; Division of Clinical Epidemiology and Aging Research, German Cancer Research Center (DKFZ), Heidelberg, Germany; Division of Preventive Oncology, German Cancer Research Center (DKFZ) and National Center for Tumor Diseases (NCT), Heidelberg, Germany; German Cancer Consortium (DKTK), German Cancer Research Center (DKFZ), Heidelberg, Germany; Department of Gastroenterology, Institut d'Investigacions Biomèdiques August Pi i Sunyer (IDIBAPS), Centro de Investigación Biomédica en Red de Enfermedades Hepáticas y Digestivas (CIBERehd), Hospital Clínic, Barcelona, Spain; Division of Gastroenterology, Massachusetts General Hospital and Harvard Medical School, Boston, MA, USA; Channing Division of Network Medicine, Brigham and Women's Hospital and Harvard Medical School, Boston, MA, USA; Clinical and Translational Epidemiology Unit, Massachusetts General Hospital and Harvard Medical School, Boston, MA, USA; Broad Institute of Harvard and MIT, Cambridge, MA, USA; Department of Epidemiology, Harvard TH Chan School of Public Health, Harvard University, Boston, MA, USA; Department of Immunology and Infectious Diseases, Harvard TH Chan School of Public Health, Harvard University, Boston, MA, USA; Department of Epidemiology and Biostatistics, University of California-San Francisco, San Francisco, CA, USA; Department of Medical Oncology & Therapeutics Research and Center for Precision Medicine, City of Hope National Medical Center, Duarte, CA, USA; Department of Radiation Sciences, Oncology Unit, Umeå University, Umeå, Sweden; Wallenberg Centre for Molecular Medicine, Umeå University, Umeå, Sweden; Public Health Sciences Division, Fred Hutchinson Cancer Center, Seattle, Washington, USA; University of Hawaii Cancer Center, Honolulu, HI, USA; Public Health Sciences Division, Fred Hutchinson Cancer Center, Seattle, Washington, USA; Department of Epidemiology, University of Washington, Seattle, WA, USA; Broad Institute of Harvard and MIT, Cambridge, MA, USA; Department of Epidemiology, Harvard TH Chan School of Public Health, Harvard University, Boston, MA, USA; Program in MPE Molecular Pathological Epidemiology, Department of Pathology, Brigham and Women's Hospital, Harvard Medical School, Boston, MA, USA; Department of Oncologic Pathology, Dana-Farber Cancer Institute, Boston, MA, USA; Department of Medicine, University of Utah, Salt Lake City, UT, USA; Genomic Medicine Institute, Cleveland Clinic, Cleveland, OH, USA; Population and Cancer Prevention Program, Case Comprehensive Cancer Center, Cleveland, OH, USA; Institute of Environmental Medicine, Karolinska Institutet, Stockholm, Sweden; University of Southern California, Preventative Medicine, Los Angeles, CA, USA; Public Health Sciences Division, Fred Hutchinson Cancer Center, Seattle, Washington, USA; Department of Epidemiology, University of Washington, Seattle, WA, USA; Department of Epidemiology and Biostatistics, Imperial College London, School of Public Health, London, UK; Nutrition and Metabolism Branch, International Agency for Research on Cancer, Lyon, France; Department of Hygiene and Epidemiology, University of Ioannina School of Medicine, Ioannina, Greece; Department of Epidemiology and Biostatistics, Imperial College London, School of Public Health, London, UK

**Keywords:** Body mass index, BMI, obesity, mediation analysis, colorectal cancer, CRC, Mendelian randomization

## Abstract

**Background:**

Colorectal cancer (CRC) is the third-most-common cancer worldwide and its rates are increasing. Elevated body mass index (BMI) is an established risk factor for CRC, although the molecular mechanisms behind this association remain unclear. Using the Mendelian randomization (MR) framework, we aimed to investigate the mediating effects of putative biomarkers and other CRC risk factors in the association between BMI and CRC.

**Methods:**

We selected as mediators biomarkers of established cancer-related mechanisms and other CRC risk factors for which a plausible association with obesity exists, such as inflammatory biomarkers, glucose homeostasis traits, lipids, adipokines, insulin-like growth factor 1 (IGF1), sex hormones, 25-hydroxy-vitamin D, smoking, physical activity (PA) and alcohol consumption. We used inverse-variance weighted MR in the main univariable analyses and performed sensitivity analyses (weighted-median, MR–Egger, Contamination Mixture). We used multivariable MR for the mediation analyses.

**Results:**

Genetically predicted BMI was positively associated with CRC risk [odds ratio per SD (5 kg/m^2^) = 1.17, 95% CI: 1.08–1.24, *P*-value = 1.4 × 10^−5^] and robustly associated with nearly all potential mediators. Genetically predicted IGF1, fasting insulin, low-density lipoprotein cholesterol, smoking, PA and alcohol were associated with CRC risk. Evidence for attenuation was found for IGF1 [explained 7% (95% CI: 2–13%) of the association], smoking (31%, 4–57%) and PA (7%, 2–11%). There was little evidence for pleiotropy, although smoking was bidirectionally associated with BMI and instruments were weak for PA.

**Conclusions:**

The effect of BMI on CRC risk is possibly partly mediated through plasma IGF1, whereas the attenuation of the BMI–CRC association by smoking and PA may reflect confounding and shared underlying mechanisms rather than mediation.

Key Messages

- Using the Mendelian randomization framework, we investigated the plausibility of putative biomarkers and other colorectal cancer (CRC) risk factors as potential mediators in the association between body mass index (BMI) and CRC.
- The effect of BMI on CRC risk is possibly partly mediated through plasma insulin-like growth factor 1 (IGF1), whereas the attenuation observed by smoking and physical activity may reflect shared underlying mechanisms and confounding rather than mediation.
- Understanding the pathophysiological pathways underlying the association between adiposity and risk of CRC may facilitate the development of targeted interventions for susceptible individuals.

## Introduction

One in 10 incident cancers diagnosed in 2020 was colorectal cancer (CRC) and its global rates are increasing.^1^ This increase may partly reflect changes in dietary and lifestyle habits, and a rise in the prevalence of excess body weight in the population, commonly captured using body mass index (BMI). Increased BMI is considered an established risk factor for CRC and 12 other cancers.^2^ Based on a World Cancer Research Fund dose–response meta-analysis, a 5-kg/m^2^ increase in BMI is associated with a 5% increase in CRC risk.^3^ Understanding the pathophysiological pathways behind the link between excess adiposity and CRC may inform the development of targeted interventions for susceptible individuals.

Epidemiological and experimental studies suggest that increased adiposity may contribute to elevated CRC risk through effects on inflammation, sex-steroid hormone and adipokine concentrations, hyperglycaemia and hyperinsulinemia, and other metabolic intermediates.^4–8^ Even though no clear consensus on which mediators explain the obesity–CRC relationship has been reached, specific patterns of mediators have emerged through observational studies. For instance, metabolic intermediates related to glucose metabolism and leptin bioavailability appear to be responsible for mediating the effects of adiposity on CRC risk more strongly than inflammatory- or sex-hormone-related markers.^5–9^

Most studies to date have used traditional methods for studying mediation in observational settings based on the model proposed by Baron and Kenny, which relies on strong and untestable assumptions (these include absence of unmeasured confounding in the associations between exposure, mediator and outcome, and absence of exposure–mediator interaction).^4–6^^,^^10^^,^^11^ Furthermore, most of these observational studies do not cover a wide range of plausible molecular pathways, typically include a small number of participants (<1000 cases in most of the studies) and are prone to biases (such as confounding).^4–10^

The Mendelian randomization (MR) framework that could overcome some of the above limitations can be used in a multivariable (MV) context, using summary genetic association estimates, allowing a mediation analysis to be performed.^12^^,^^13^ In the present study, we use the MR framework to: (i) investigate the plausibility of putative biomarkers and other CRC risk factors as potential mediators in the association between BMI (as a measure of general adiposity) and CRC risk, and (ii) to estimate the proportion of the mediated effect explained by plausible mediators.

## Methods

### Source of genetic instruments

We selected as potential mediators biomarkers of established cancer-related mechanisms for which a plausible association with BMI exists based on epidemiological and experimental evidence.^14^ These potential mediators included inflammation-related biomarkers [C-reactive protein (CRP) and interleukin-6 (IL6)], metabolism-related biomarkers [glucose homeostasis, lipids, adipokines, insulin-like growth factor 1 (IGF1)], sex hormones and 25-hydroxy-vitamin D [25(OH)D].^3^^,^^14^ We also included CRC risk factors that typically act as confounders, such as smoking, physical activity (PA), alcohol consumption and type 2 diabetes, in the analysis, to examine the possibility of mediation vs confounding (Supplementary Figure S1, available as Supplementary data at *IJE* online).^15^

Details of the genome-wide association studies (GWASs) from which we selected the genetic instruments are presented in the Supplementary Methods, Supplementary Table S1 (available as Supplementary data at *IJE* online) and in the originally published studies.

Summary genetic association estimates for CRC, as well as cancers of the colon, proximal and distal colon and rectum, and in men and women separately, were obtained from a GWAS meta-analysis of the Genetics and Epidemiology of Colorectal Cancer Consortium (GECCO), Colorectal Transdisciplinary Study (CORECT) and Colon Cancer Family Registry (CCFR)^16^ (Supplementary Table S2, available as Supplementary data at *IJE* online).

### Instrument selection and MR analyses

In the univariable MR analyses, we selected as instruments single-nucleotide polymorphisms (SNPs) that were associated with the exposure of interest (BMI and the putative mediators) at genome-wide significance (*P *<* *5 × 10^−8^), allowing a weak linkage disequilibrium (*r*^2^ < 0.001) (Supplementary Table S3, available as Supplementary data at *IJE* online). The random-effects inverse-variance weighted (IVW) model was used as the main analysis and the MR–Egger, Contamination Mixture and weighted-median MR methods were used as sensitivity analyses.^17–19^ These analyses were used to investigate the association of genetically predicted BMI on CRC, mediators on CRC and BMI on mediators bidirectionally. Bidirectional analyses between BMI and mediators were performed to examine confounding vs mediation and to gain a deeper insight into the main associations. Mediators for which a ‘robust’ association was found in the univariable analysis with BMI (BMI to mediators) and CRC (mediators to CRC) were included in the subsequent mediation analyses (Supplementary Methods, available as Supplementary data at *IJE* online). For discrepant BMI-to-mediator and mediator-to-CRC associations, clustered heterogeneity in the BMI-to-mediator association was -assessed using an expectation–maximization-based model fitting, to identify potentially distinct pathways that would explain the observed discrepancy.^20^

In the multivariable MR (MVMR) analyses, we used a combined genetic instrument, selecting uncorrelated variants (*r*^2^<0.001) associated with any of the exposures in the model. The genetic association estimates for CRC were regressed on the genetic association estimates for BMI and mediator(s), weighted for the precision of the genetic association estimates for CRC and with the intercept fixed to zero.^21^ Similarly, we performed MV MR–Egger analyses, allowing an intercept, as an indicator of directional pleiotropy.

### Mediation analysis

Univariable MR analyses were used to estimate the total effect of genetically predicted BMI on CRC risk. MVMR analyses were used to assess mediation and correction for horizontal pleiotropy. Using MVMR models, adjusting for one mediator at a time, the direct effects of BMI on CRC risk was estimated (i.e. the effect of BMI on CRC through pathways other than the mediator in the model). The indirect effect of BMI on outcome was estimated using the difference in coefficients method, which was expressed as the proportion attenuated.^12^^,^^13^

**Figure 1.**
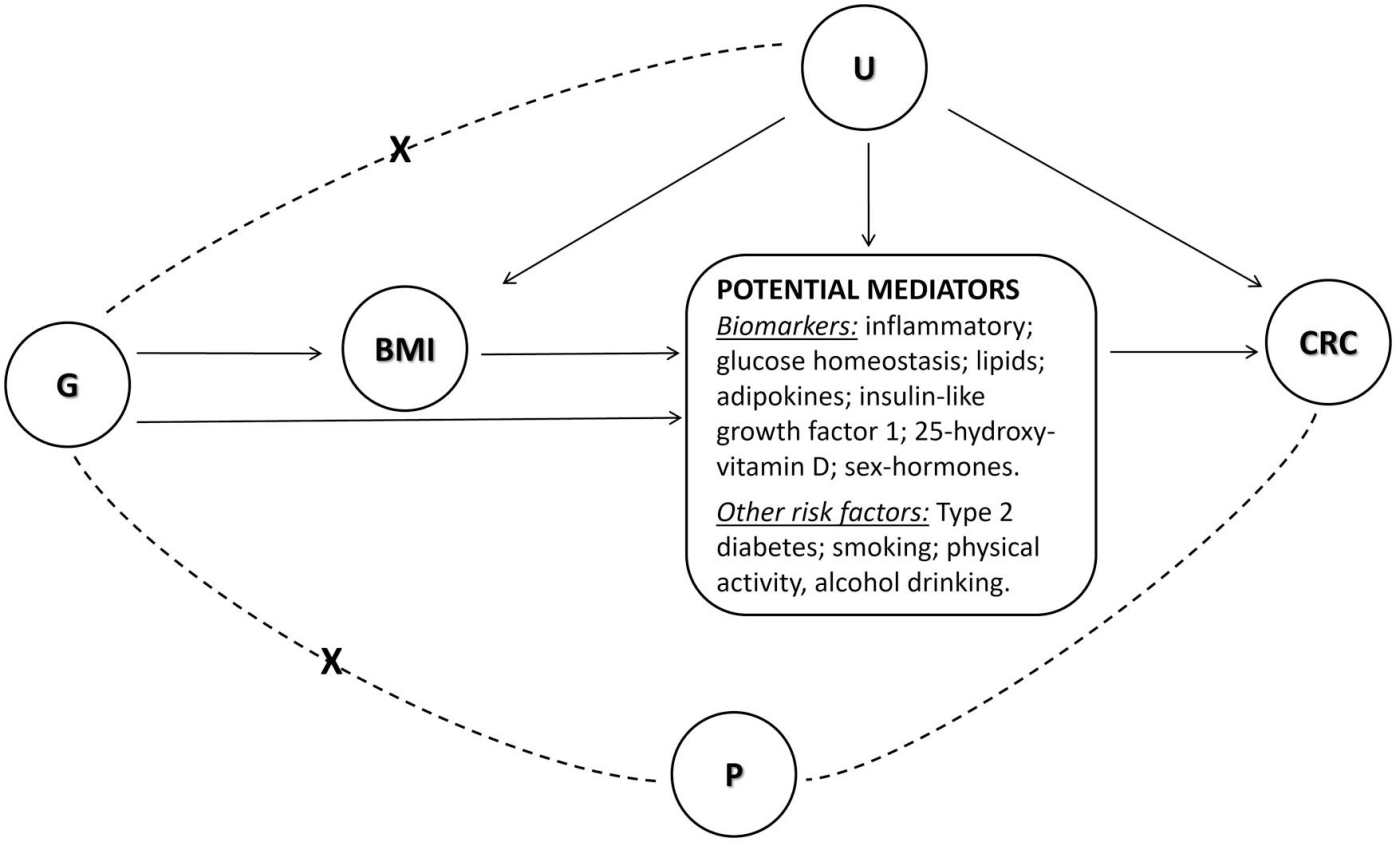
To obtain valid causal estimates for mediation, the three typical Mendelian randomization (MR) assumptions should be satisfied in the context of multivariable MR. These are: (i) relevance—the single-nucleotide polymorphisms (SNPs) (G) should be strongly associated with body mass index (BMI) [given the putative mediator(s) included in the model]; (ii) exchangeability—the SNPs (G) should be independent of all confounders (U) of any of the exposures (in the model) and colorectal cancer (CRC) and (iii) exclusion restriction—there should be no other path (P) through which the SNPs affect CRC but via BMI [or the putative mediator(s) in the model]

To investigate the validity of the three core MR assumptions (Figure 1), in the context of MVMR: (i) relevance [the SNPs should be strongly associated with BMI given the mediator(s) included in the model], (ii) exchangeability (the SNPs should be independent of confounders of any of the exposures in the model and CRC) and (iii) exclusion restriction [there should be no other path through which the SNPs affect CRC but via BMI, or the mediator(s) in the model], several approaches were used (Supplementary Methods, available as Supplementary data at *IJE* online).^13^ Briefly, we used the conditional F-statistic (*F*_cond_) as an indicator of instrument strength.^22^ For mediators with conditionally weak instruments (*F*_cond_<10), we reran the MVMR and mediation analyses using alternative instruments for BMI as a means of enhancing the conditional strength of the mediator(s) in the model. Sensitivity analyses were performed excluding genetic instruments significantly associated with both BMI and mediator in the MV model, as a means of distinguishing the mediation phenomenon from horizontal pleiotropy.^13^ The Phenoscanner database was used to explore the previously reported associations of the selected genetic instruments and identify potentially pleiotropic pathways.

In secondary analyses, we investigated the mediation on the subtypes of CRC, and among men and women separately. All analyses were performed using R (v.4.1.1).^23^

## Results

### Evaluating the effect of BMI on CRC (total effects)

**Figure 2.**
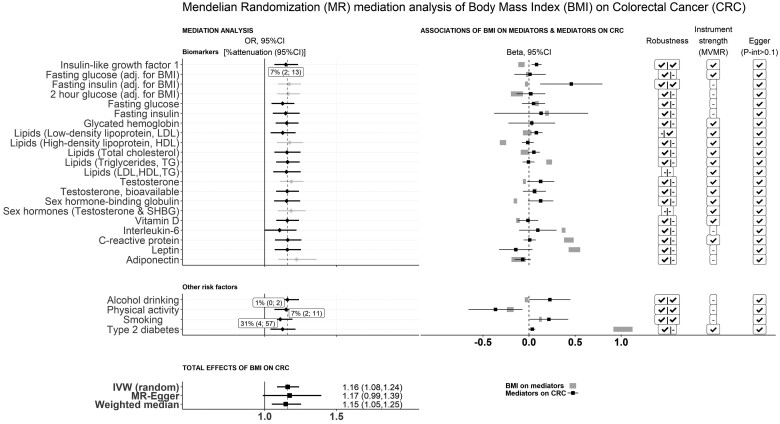
Mendelian randomization (MR) mediation analysis of genetically predicted body mass index (BMI) on colorectal cancer (CRC) risk. The forest plot (left, top and middle) presents the MR inverse-variance weighted (IVW) estimate (OR, 95% CI) of BMI on CRC risk in the MVMR model, adjusted for one putative mediator at a time (direct effects). Where there was a robust BMI-to-putative-mediator and mediator-to-CRC-risk association, the percent attenuation and 95% CI were estimated and are presented in labels. The forest plot on the right side displays the marginal associations of BMI on putative mediators (the 95% CI of the MR–IVW using a thick grey line) and mediators on CRC risk (the MR–IVW beta and 95% CI). The check marks in the first column (‘Robustness’) indicate robust marginal associations [i.e. significant associations in the IVW analysis (*P *<* *0.05) that were qualitatively consistent in sensitivity analyses], in the second column [‘Instrument strength (MVMR)’] indicate conditional F-statistics of >10 [for both predicted body mass index (BMI) and the putative mediator in the model] and in the third column (‘Egger P-int.>0.1’) indicate that the *P*-value of the intercept in the MV MR–Egger models was >0.1. The forest plot at the bottom displays the total effects (OR, 95% CI) of BMI on CRC risk in univariable MR–IVW models

**Figure 3.**
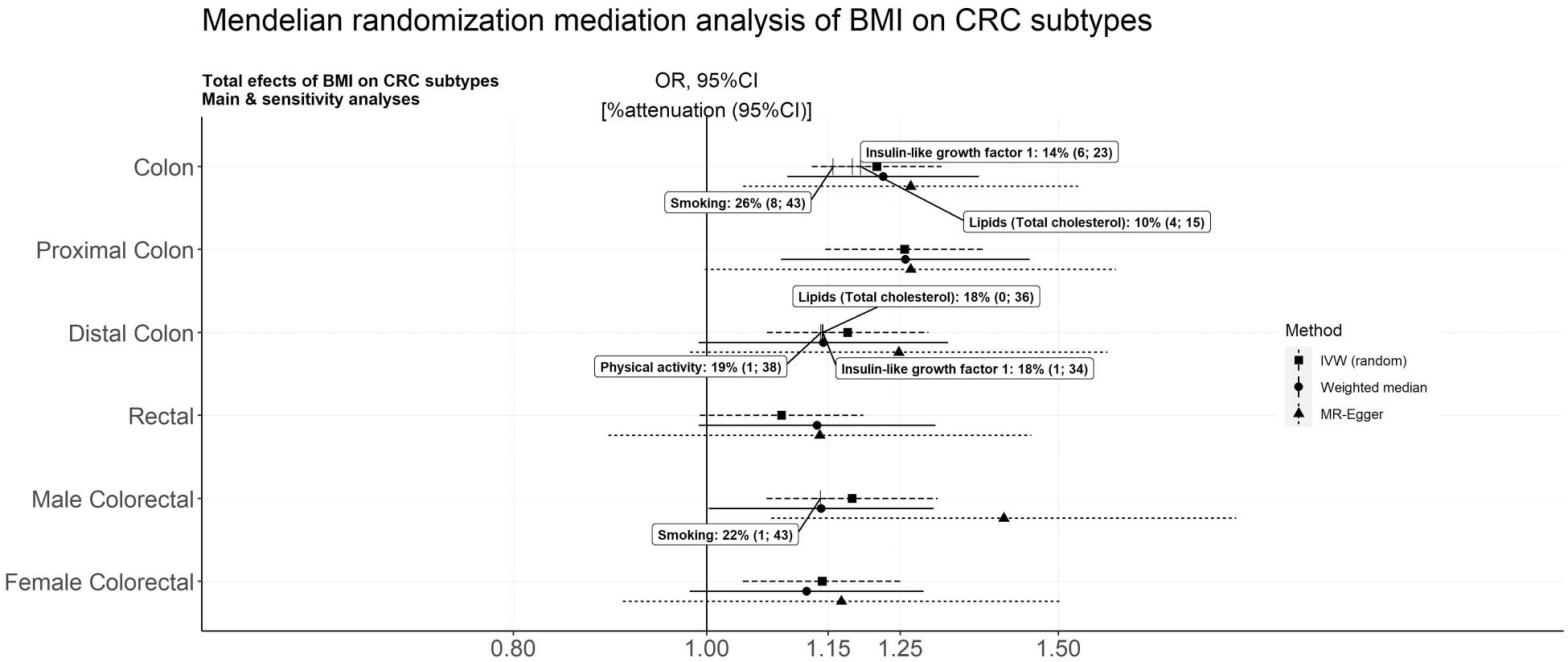
Mendelian randomization (MR) mediation of genetically predicted body mass index (BMI) on colorectal cancer (CRC) subtypes. The forest plot shows the OR (95% CI) per SD of genetically predicted BMI (5 kg/m^2^), on CRC subtype in the MR inverse-variance weighted (IVW) models (total effects) and in sensitivity analyses. Where there was a robust BMI-to-putative-mediator and mediator-to-CRC-risk association, the percent attenuation and 95% CI were estimated and are presented in labels

There was consistent evidence across the IVW and sensitivity analyses that higher genetically predicted BMI increased overall CRC risk [odds ratio (OR) per SD (4.7 kg/m^2^) higher BMI: 1.16, 95% CI: 1.08–1.24, *P* = 1.4×10^−6^] (Figure 2 and Supplementary Table S4, available as Supplementary data at *IJE* online). A positive effect of BMI remained in the analyses by CRC subtypes and by sex. It was marginally stronger for colon vs rectal cancer (*P*_het_=0.07) but similar for proximal vs distal colon cancer (*P*_het_=0.32) and by sex (*P*_het_=0.58) (Figure 3 and Supplementary Table S4, available as Supplementary data at *IJE* online**)**.

### Evaluating the effect of mediators on CRC

There was consistent evidence across the IVW and sensitivity analyses that CRC risk was associated with genetically predicted IGF1 (OR per 1 unit in rank-based inverse normal transformed IGF1, which approximates to 5.7 nmol/L: 1.08, 95% CI: 1.03–1.15), fasting insulin (OR per 1 unit in natural log-transformed pmol/L: 1.58, 95% CI: 1.13–2.22), low-density lipoprotein (LDL) cholesterol (OR per 1 unit increase in rank-based inverse normal transformed LDL cholesterol: 1.08, 95% CI: 1.01–1.16), smoking (OR per 1 unit of lifetime smoking index, which approximates to 20 cigarettes/day for 10 years: 1.24, 95% CI: 1.01–1.53), PA {OR per SD [0.08 m/s^2^, which approximates to 3 units of metabolic equivalent task score (METS)-h/day]: 0.70, 95% CI: 0.52–0.93} and alcohol drinking [OR per SD (∼5.6 drinks/week): 1.25, 95% CI: 1.00–1.57]. Furthermore, T2DM was positively associated with CRC (OR per 1-unit increase in log odds for T2DM liability: 1.04, 95% CI: 1.00–1.07), albeit the association was not consistent in sensitivity analyses (Supplementary Table S5, available as Supplementary data at *IJE* online).

### Evaluating the effect of BMI on mediators

Using MR–IVW analyses, we found that genetically predicted BMI was positively associated with HbA1c, fasting glucose and insulin (using the instrument unadjusted for BMI), T2DM liability, triglycerides, bioavailable testosterone, oestradiol among men, IL6, CRP, leptin and smoking, and inversely associated with IGF1, fasting glucose and insulin, and two-h glucose (2hGlu) (using instruments adjusted for BMI, likely due to collider bias), HDL-C, total cholesterol, total testosterone, sex hormone binding globulin (SHBG), 25(OH)D, adiponectin, PA and alcohol drinking. The above associations were largely consistent in the sex-stratified analyses, except for the associations of BMI with total testosterone and bioavailable testosterone, which were inverse in men and positive in women (Supplementary Table S6, available as Supplementary data at *IJE* online).

### Evaluating the mediator-to-mediator associations

Several mediator-to-mediator associations, consistent in sensitivity analyses, were found in the univariable MR analyses. Evidence of bidirectionality with BMI was found for fasting insulin and 2hGlu (using instruments adjusted for BMI), total cholesterol and smoking (Supplementary Figure S2 and Supplementary Table S7, available as Supplementary data at *IJE* online).

### MR mediation analysis

IGF1 was found to be a plausible mediator (7% of total effects mediated, 95% CI: 2–13%, *P *=* *0.01) in the association of genetically predicted BMI with overall CRC risk, with little evidence of residual directional pleiotropy or weak instrument bias (*F*_cond_=46) (Figure 2 and Supplementary Table S8, available as Supplementary data at *IJE* online).

Additionally, attenuation of the effects of BMI on CRC risk was found for smoking (31%, 95% CI: 4–57%, *P *=* *0.02), PA (7%, 2–11%, *P *=* *2 × 10^−3^) and alcohol drinking (1%, 0–2%, *P *=* *0.05). However, the conditional F-statistics were low, indicating weak instruments (*F*_cond_ range: 2–7) (Figure 2 and Supplementary Table S8, available as Supplementary data at *IJE* online). When we used alternative instruments for BMI, the results did not change materially (Supplementary Table S9, available as Supplementary data at *IJE* online).

In sensitivity analysis excluding genetic instruments significantly associated with both BMI and the mediator in the model, the results did not change materially (Supplementary Table S10 and Supplementary Figure S3, available as Supplementary data at *IJE* online).

We found no major pleiotropic pathways (other than those included as mediators), apart from a few SNPs previously associated with inflammatory bowel disease (Supplementary Tables S11 and S12, available as Supplementary data at *IJE* online) that are unlikely to have substantially influenced the mediation estimation. Discrepant associations were found for IGF1 and alcohol, with evidence of clustered heterogeneity in the association of BMI with both mediators, supporting the presence of distinct pathways, potentially inferring positive and inverse effects (Supplementary Table S13 and Supplementary Figure S4, available as Supplementary data at *IJE* online).

In the secondary analyses performed by subtype and sex, evidence for mediation was found for IGF1, total cholesterol and smoking in relation to colon cancer; IGF1, total cholesterol and PA for distal colon cancer; and smoking for CRC in men (Figure 3 and Supplementary Table S8, available as Supplementary data at *IJE* online). However, most of these analyses were based on smaller samples and the power was limited.

## Discussion

Using MR analyses, we investigated the mediating effects of several biologically plausible intermediates in the association between BMI and CRC. Our results suggest that some of the effects of genetically predicted BMI on CRC and subtypes is mediated through IGF1. Fasting insulin was also a plausible mediator, robustly associated with BMI and CRC risk, although no mediating effect was found, possibly due to the weak instrument for insulin. Attenuation of the effects of BMI on CRC risk was also observed by smoking and PA, although these exposures typically act as confounders or common underlying factors, hence adjustment via MVMR likely provides a reflection of adjustment for horizontal pleiotropy. The bidirectional association of BMI with (smoking) and the shared carcinogenic mechanisms for BMI and PA strengthen the notion that these factors are less likely to act as mediators.

We found that higher genetically predicted BMI was associated with decreased IGF1 concentrations and that IGF1 was positively associated with CRC risk. Such a discrepancy in the direction of the marginal associations might suggest that BMI acts to increase CRC risk via IGF1, via complex modes of effect that potentially involve other intermediates (such as insulin).^24^ Such a complexity is underscored by clusters of variants influencing exposures and mediators in diverse ways, potentially targeting distinct causal effect parameters, and is partly addressed by using the composite instruments. Nevertheless, such a complexity may not be sufficiently represented by individual biomarkers, but rather require patterns of biomarkers, which, however, were not captured in our analyses, partly due to the limited power that exists with multiple mediators within the MVMR framework. Though not in universal agreement, epidemiological studies support an inverse association of BMI with circulating IGF1 levels.^25^^,^^26^ Such an inverse association might be explained by the presence of a negative feedback loop among individuals with a prolonged state of obesity, whereby reductions in IGF binding protein 1 (IGFBP1) and IGF binding protein 2 (IGFBP2), as a result of obesity-associated hyperinsulinemia, may lead to increased negative feedback by free IGF1 (unbound to IGFBPs) on pituitary growth hormone secretion (a major regulator of IGF1 synthesis in the liver).^27^ Furthermore, time-varying effects of BMI on the concentrations of IGF1 have been previously reported, which could also explain the observed discrepancy in the direction of the BMI-to-IGF1 association. For instance, a cross-sectional study (*n* = 4241 participants from the Long Life Family Study) found evidence of an inverse association among individuals in the lowest age groups (20–66 years old) and a positive association among older individuals (>87 years old).^28^ Prospective studies have shown that increased concentrations of circulating IGF1 are positively associated with CRC risk—an association that has been replicated in previous MR analyses and is concordant with our results.^29^^,^^30^ In support of the observed positive association, a large body of mechanistic evidence has shown that IGF1 has proliferative and anti-apoptotic effects and activates cellular pathways linked to tumorigenesis such as the PI3K-mTOR and RAS pathways.^31–33^ A recent observational study investigating the contribution of metabolic mediators in the association between adult weight gain and CRC found no evidence of mediation for IGF1, but the study was small, including 266 colon and 186 rectal cancer cases.^5^

A relatively strong attenuation of the effects of BMI on CRC risk was observed for smoking. Causal associations of BMI on smoking and of smoking on CRC have been previously reported and confirmed in our analysis.^34^^,^^35^ Purported mechanisms through which BMI is linked to smoking are complex and most likely have behavioural and metabolic implications.^36^ Higher BMI and tobacco smoking genetically share a biological basis for addictive behaviours, such as nicotine addiction and higher energy intake.^34^ Higher BMI has been related to lower socio-economic status, income and educational attainment, and lower educational attainment may lead to increased smoking, whereas obesity may increase nicotine dependence and affect smoking intensity.^37^^,^^38^ Tobacco smoking as a source of a multitude of carcinogenic compounds, likely acting via their immunosuppressive effects, which can play an important tumour-promoting role in the serrated neoplasia pathway (characterized by high-level microsatellite instability and vigorous immune response), has been well established as a CRC risk factor.^39^ We found a bidirectional (positive) association of genetic predisposition to higher lifetime amount of smoking on BMI, which supports the hypothesis that smoking may act as a confounder. Such a positive association (of smoking on BMI) finds little support in previously published observational or MR analyses.^36^^,^^40^

There is ample epidemiological evidence suggesting that obesity in childhood is associated with reduced PA, but research on adult populations is largely focused on the effectiveness of PA as a means of maintaining a healthy weight.^41^^,^^42^ Previous observational and MR analyses provided evidence to support an association between BMI and PA levels, whereas a bidirectional association with BMI has also been reported.^43–45^ We found an inverse association between genetically predicted BMI and device-measured PA, but not vice-versa, which could be attributed to the relatively weak instrument for device-measured PA. Furthermore, higher genetically predicted PA was associated with a 30% decreased CRC risk, in line with previous studies.^43^ Unhealthy lifestyle behaviours (such as sedentary behaviour, time spent watching television) that accompany obesity might impact an individual’s levels of PA.^46^ On the other hand, increased PA may act to reduce CRC risk via multiple biological mechanisms that include improvement in insulin sensitivity and inflammation, digestion stimulation and transit time stimulation in the intestine.^47^ The fact that BMI and PA share multiple carcinogenic mechanisms questions the notion that PA in fact acts as a mediator in the association between BMI and CRC risk.

In this large-scale MR analysis, we included a wide panel of biologically plausible mediators covering different mechanisms and the largest samples available to date for the exposure, mediators and outcomes. We acknowledge that our study has several limitations that should be considered when interpreting the results. The present analysis is based on BMI as a measure of general adiposity, which does not capture the type of adiposity and the proportion relative to lean mass; nevertheless, BMI is highly correlated with objective measures of adiposity and, considering its wide use in the epidemiological setting (because it is relatively easy and straightforward to measure), large GWAS sample sizes have been achieved and hence power is increased compared with other measures of adiposity (such as radiology-based measures).^48^ Several potentially important pathways or pathway-specific traits, such as detailed inflammation-related intermediates or adipokines, were not included (or inadequately represented in our panel of mediators) due to absence of valid instruments. This also limited our ability to identify patterns of biomarkers, which could elucidate the complex pathways in which higher BMI is associated with increased CRC risk. The total effects of BMI on CRC risk were relatively small, which might have caused the precision in the estimate of the indirect effects and the proportion attenuated to inflate. There was no distinction between childhood and adulthood BMI; however, a recent analysis demonstrated that most of the effects of the childhood BMI on CRC are mediated through adulthood BMI.^49^ Time-varying effects of BMI on some biomarkers (such as IGF1) have been reported, which could be a threat to the validity of the mediating estimates and should be explored in future MR mediation analyses using individual-level data.^28^ Measurement errors may not be of utmost concern in the present analysis, although we cannot rule out the possibility that intra-individual variation for some of the included traits may have affected the validity of the estimates to some extent.^50^^,^^51^ Finally, the analysis was based on individuals of European descent and there was limited power to expand the mediation analysis to populations of different racial and ethnic groups.

## Conclusions

In conclusion, the effects of BMI on CRC risk may be partly mediated through IGF1. Future studies using stronger instruments for plausible intermediates are warranted to more comprehensively unveil the mechanisms linking adiposity to CRC risk. The complex nature of the causal associations of the intermediates highlights the need to consider the major risk factors together in the development of CRC prevention strategies.

## Disclaimer

Where authors are identified as personnel of the International Agency for Research on Cancer/World Health Organization, the authors alone are responsible for the views expressed in this article and they do not necessarily represent the decisions, policy or views of the International Agency for Research on Cancer/World Health Organization.

## Ethics approval

Not applicable.

## Supplementary Material

dyae067_Supplementary_Data

## Data Availability

All data used in this work are presented in the Additional files that accompany the manuscript and are available in the original publications.
