# Supplementary material for: Identification of potential mediators of the relationship between body mass index and colorectal cancer: a Mendelian randomization analysis
Source: Int J Epidemiol. 2024 May 9;53(3):dyae067. doi: 10.1093/ije/dyae067 (PMC11082423; doi:10.1093/ije/dyae067)
Supplement: dyae067_Supplementary_Data [file dyae067_supplementary_data.zip › ije-2023-09-1159-File005.pdf]

Supplementary File

Identification of potential mediators of the relationship between body mass index and colorectal cancer: a Mendelian randomization analysis

Content:

Supplementary Methods

Supplementary Methods

Source of genetic instruments

We selected as potential mediators biomarkers of established cancer-related mechanisms, for which a plausible association with obesity exists based on epidemiological and experimental evidence. These potential mediators included inflammation-related biomarkers [(C-reactive protein (CRP), as a non-specific marker of inflammation and interleukin-6 (IL6)], metabolism-related biomarkers [glucose homeostasis, lipids, adipokines, insulin-like growth factor 1 (IGF1)], sex-hormones, and 25-hydroxy-vitamin D (25(OH)D) [1,2].

Other CRC risk factors, such as smoking, physical activity (PA), and alcohol, are associated with BMI (and probably some of the above biomarkers) and CRC, and typically act as confounders. However, previous studies showed that BMI might have causal effects on these factors, supporting that notion that they might as well act as intermediates in the causal path that links BMI to CRC risk [3,4]. To examine the possibility of mediation versus confounding we included the above risk factors among the potential mediators (***Supplementary*** **Figure 1**) [5].

A detailed description of the GWAS studies from which we selected the genetic instruments can be found in the ***Supplementary Table 1*** and in the originally published studies. In brief, genetic association estimates for BMI were obtained from a GIANT Consortium GWAS meta-analysis of 806 834 individuals [6]. Estimates for genetic liability to type 2 diabetes (T2DM) were obtained from a DIAGRAM Consortium GWAS of 74 124 cases and 824 006 controls, and estimates for fasting glucose (FG), fasting insulin (FI), two-hour glucose (2hGlu), and HbA1c were obtained from a GWAS of up to 200 622 non-diabetic individuals [7,8]. Since estimates for the latter biomarkers (FG, FI, and 2hGlu) were produced in models adjusted for BMI, we also used instruments from alternative sources (available for FG and FI) to avoid potential collider bias in

the MR analyses (adjusting for BMI in the initial models might induce false positive genetic association estimates for SNPs that are associated with BMI) [9]. Genetic association estimates for circulating CRP and IL6 (cis-instruments based on the CRP pathway as previously described [10]) were obtained from a GWAS of up to 575 531 individuals [11] . Genetic association estimates for fasting serum lipids, namely low-density lipoprotein cholesterol (LDL-C), high-density lipoprotein cholesterol (HDL-C), total cholesterol (T-C), and triglycerides (TG) were obtained from a Global Lipids Genetic Consortium GWAS including up to 1 320 000 individuals [12]. Estimates for adiponectin were obtained from a GWAS of up to 65 521 individuals [13]. Instruments for leptin were extracted from a SCALLOP Consortium GWAS MA of up to 30 931 individuals [14]. Estimates for alcohol were obtained from a GWAS and Sequencing Consortium of Alcohol and Nicotine use (GSCAN) GWAS including 941 280 individuals [15]. Genetic association estimates for IGF1, estradiol, testosterone, bio-available testosterone, sex-hormone binding globulin (SHBG), 25(OH)D, device-measured physical activity (PA) and a lifetime smoking index (capturing the lifetime exposure to smoke) were obtained from GWAS studies of up to 462 690 European-ancestry individuals in the UK Biobank [16-19].

All the above GWAS studies included only European ancestry individuals, except for the study for adiponectin (which included approximately 11.1% non-Europeans). For BMI, adiponectin, 25(OH)D, SHBG, estradiol (men only), testosterone (total and bioavailable), T2DM, fasting glucose and insulin, and IGF1, sex-specific instruments were available, which were used in all of the sex-stratified MR analyses.

Summary genetic association estimates for overall and site-specific CRC, namely cancers of the colon, rectum, proximal and distal colon, and in men and women separately, in up to 58 221 cases and 67 694 controls were obtained from an analysis of the Genetics and Epidemiology of

Colorectal Cancer Consortium (GECCO), Colorectal Transdisciplinary Study (CORECT), and Colon Cancer Family Registry (CCFR) [20]. The distribution of cases and controls per individual study are presented in ***Supplementary Table 2.***

Instrument selection & Mendelian Randomization analyses

In the univariable (UV) MR analyses, we selected as instruments single nucleotide polymorphisms (SNPs) that were associated with the exposure of interest (BMI and the mediators) at genome-wide significance ($P<5x10^{-8}$) and in weak linkage disequilibrium with each other ($r^2<0.001$) (***Supplementary Table 3***). The random-effects inverse-variance weighted (IVW) was used as the main analysis and the MR-Egger, Contamination-Mixture and Weighted Median MR methods were used as sensitivity analyses [21-23]. These analyses were used to investigate the potential causal association of BMI on CRC risk, mediators on CRC risk, and BMI on mediators bidirectionally.

Significant associations in the IVW analysis ($P<0.05$) that were qualitatively consistent in all sensitivity analyses (all betas in the same direction and either of the following being true: MR-Egger P-value <0.2 or Weighted Median P-value <0.1 or Contamination-Mixture P-value <0.1) were considered ‘robust’ in all MR analyses.

In the multivariable MR (MVMR) analyses, we used a combined genetic instrument, selecting uncorrelated variants ($r^2<0.001$) associated with any of the covariates in the model [i.e., BMI and mediator(s)], using a pairwise P-value threshold of $5x10^{-8}$ across covariates. The genetic association estimates for CRC were regressed on the genetic association estimates for BMI and mediator(s), weighted for the precision of the genetic association estimates for CRC, and with the intercept fixed to zero [24]. Similarly, we performed MV MR-Egger analyses, allowing for an intercept, as an indicator of directional pleiotropy. All mediators were considered individually in MV models, and the lipids (LDL-C, HDL-C and TG), and the sex-hormones were considered

simultaneously in MV models. Throughout the analyses, the instrument effect alleles were aligned according to the main exposure.

Mediation analysis

The total effect of BMI on CRC was estimated as the association of the genetically predicted BMI on CRC risk in the UV MR analysis. Using MVMR models, the direct effects of BMI on risk of CRC (i.e., the effect of BMI on CRC through pathways other than the mediator in the model) was estimated adjusting for one mediator at a time. Mediators for which a *robust* association was found in the UV analysis with BMI (with BMI as the exposure) and CRC (with the mediator as the exposure) were included in the subsequent mediation analyses. Where there was evidence for 'robust' marginal associations of BMI to mediator and mediator to CRC, the indirect effect of BMI on outcome was estimated using the difference in coefficients method (subtracting the direct effects from total effects), along with the percentage of attenuation (i.e., the proportion of the effect of BMI on risk of CRC that was mediated through the mediator) [25]. In secondary analyses, the product of coefficients method was used to estimate the indirect effects (multiplying the beta-coefficient of the exposure-mediator association by the beta-coefficient of the mediator-outcome association from the MV MR model) and the proportion mediated [26]. The propagation of errors was used to estimate the 95%CI of the proportion mediated [27].

To obtain valid causal estimates for mediation, the three MR assumptions in the context of MVMR should be met: (i) the SNPs should be strongly associated with BMI (given the mediator(s) included in the model), (ii) the SNPs should be independent of all confounders of any of the exposures and the outcome, and (iii) there should be no other path through which the SNPs affect the outcome but via BMI (or the mediator(s) in the model [26,28]. To evaluate the robustness of the associations to potential violations of the MR assumptions we used the following approaches: We

used the conditional F-statistic (F_{cond}) of the variables in the MV models as an indicator of instrument strength [29]. In sensitivity analyses, for mediators with conditionally weak instruments (F_{cond} <10), we re-run the MVMR and mediation analyses using a fraction of the BMI instruments (selecting the strongest ones, using a stricter P-value threshold of 5×10^{-14} which resulted in the retention of approximately 40% of the total instruments), as a means of enhancing the conditional strength of the mediator relative to BMI, and evaluate the consistency of the associations. Sensitivity analyses were performed excluding genetic instruments significantly associated ($P<10^{-6}/10^{-8}$) with both BMI and mediator in the MV model, as a means of distinguishing the mediation phenomenon from horizontal pleiotropy [28]. Additionally, we used the *phenoscanner* database to explore the previously reported associations of the selected genetic instruments and identify potentially pleiotropic pathways.

In secondary analyses we investigated the mediation on the subtypes of CRC, and among men and women separately.

All analyses were performed using R (v.4.1.1), and the *ieugwasr, MendelianRandomization, TwoSampleMR* and *MVMR* packages [30-33].

References

1. World Cancer Research Fund/American Institute for Cancer Research. Continuous Update Project Expert Report 2018. The cancer process. Available at: dietandcancerreport.org.
2. World Cancer Research Fund/American Institute for Cancer Research. Continuous Update Project Expert Report 2018. Diet, nutrition, physical activity and colorectal cancer. Available at dietandcancerreport.org.
3. Taylor AE, Richmond RC, Palviainen T, et al. The effect of body mass index on smoking behaviour and nicotine metabolism: a Mendelian randomization study. *Human molecular genetics* 2019; **28**: 1322-30.
4. Carrasquilla GD, García-Ureña M, Fall T, Sørensen TIA, Kilpeläinen TO. Mendelian randomization suggests a bidirectional, causal relationship between physical inactivity and obesity. *bioRxiv* 2021: 2021.06.16.448665.
5. MacKinnon DP, Krull JL, Lockwood CM. Equivalence of the mediation, confounding and suppression effect. *Prevention science : the official journal of the Society for Prevention Research* 2000; **1**: 173-81.
6. Pulit SL, Stoneman C, Morris AP, et al. Meta-analysis of genome-wide association studies for body fat distribution in 694 649 individuals of European ancestry. *Human molecular genetics* 2019; **28**: 166-74.
7. Mahajan A, Taliun D, Thurner M, et al. Fine-mapping type 2 diabetes loci to single-variant resolution using high-density imputation and islet-specific epigenome maps. *Nature genetics* 2018; **50**: 1505-13.
8. Chen J, Spracklen CN, Marenne G, et al. The trans-ancestral genomic architecture of glycemic traits. *Nature genetics* 2021; **53**: 840-60.
9. Lagou V, Mägi R, Hottenga JJ, et al. Sex-dimorphic genetic effects and novel loci for fasting glucose and insulin variability. *Nature communications* 2021; **12**: 24.
10. Georgakis MK, Malik R, Gill D, Franceschini N, Sudlow CLM, Dichgans M. Interleukin-6 Signaling Effects on Ischemic Stroke and Other Cardiovascular Outcomes: A Mendelian Randomization Study. *Circulation Genomic and precision medicine* 2020; **13**: e002872.
11. Said S, Pazoki R, Karhunen V, et al. Genetic analysis of over half a million people characterises C-reactive protein loci. *Nature communications* 2022; **13**: 2198.
12. Graham SE, Clarke SL, Wu KH, et al. The power of genetic diversity in genome-wide association studies of lipids. *Nature* 2021; **600**: 675-9.
13. Spracklen CN, Karaderi T, Yaghootkar H, et al. Exome-Derived Adiponectin-Associated Variants Implicate Obesity and Lipid Biology. *American journal of human genetics* 2019; **105**: 15-28.
14. Folkersen L, Gustafsson S, Wang Q, et al. Genomic and drug target evaluation of 90 cardiovascular proteins in 30,931 individuals. *Nature metabolism* 2020; **2**: 1135-48.
15. Liu M, Jiang Y, Wedow R, et al. Association studies of up to 1.2 million individuals yield new insights into the genetic etiology of tobacco and alcohol use. *Nature genetics* 2019; **51**: 237-44.
16. NealeLab. *UK Biobank GWAS Results*. 2018 [cited; Available from: http://www.nealelab.is/uk-biobank/
17. Doherty A, Smith-Byrne K, Ferreira T, et al. GWAS identifies 14 loci for device-measured physical activity and sleep duration. *Nature communications* 2018; **9**: 5257.
18. Wootton RE, Richmond RC, Stuijfzand BG, et al. Evidence for causal effects of lifetime smoking on risk for depression and schizophrenia: a Mendelian randomisation study. *Psychological medicine* 2020; **50**: 2435-43.
19. Ruth KS, Day FR, Tyrrell J, et al. Using human genetics to understand the disease impacts of testosterone in men and women. *Nature medicine* 2020; **26**: 252-8.
20. Huyghe JR, Bien SA, Harrison TA, et al. Discovery of common and rare genetic risk variants for colorectal cancer. *Nature genetics* 2019; **51**: 76-87.

21. Burgess S, Butterworth A, Thompson SG. Mendelian randomization analysis with multiple genetic variants using summarized data. *Genetic epidemiology* 2013; **37**: 658-65.
22. Burgess S, Foley CN, Allara E, Staley JR, Howson JMM. A robust and efficient method for Mendelian randomization with hundreds of genetic variants. *Nature communications* 2020; **11**: 376.
23. Bowden J, Davey Smith G, Haycock PC, Burgess S. Consistent Estimation in Mendelian Randomization with Some Invalid Instruments Using a Weighted Median Estimator. *Genetic epidemiology* 2016; **40**: 304-14.
24. Burgess S, Thompson SG. Multivariable Mendelian randomization: the use of pleiotropic genetic variants to estimate causal effects. *American journal of epidemiology* 2015; **181**: 251-60.
25. Burgess S, Daniel RM, Butterworth AS, Thompson SG. Network Mendelian randomization: using genetic variants as instrumental variables to investigate mediation in causal pathways. *International journal of epidemiology* 2015; **44**: 484-95.
26. Sanderson E. Multivariable Mendelian Randomization and Mediation. *Cold Spring Harbor perspectives in medicine* 2021; **11**.
27. Burgess S, Thompson DJ, Rees JMB, Day FR, Perry JR, Ong KK. Dissecting Causal Pathways Using Mendelian Randomization with Summarized Genetic Data: Application to Age at Menarche and Risk of Breast Cancer. *Genetics* 2017; **207**: 481-7.
28. Carter AR, Sanderson E, Hammerton G, et al. Mendelian randomisation for mediation analysis: current methods and challenges for implementation. *Eur J Epidemiol* 2021; **36**: 465-78.
29. Sanderson E, Spiller W, Bowden J. Testing and correcting for weak and pleiotropic instruments in two-sample multivariable Mendelian randomization. *Statistics in medicine* 2021; **40**: 5434-52.
30. Hemani G. *ieugwasr: R interface to the IEU GWAS database API*. 2020 [cited; Available from: https://github.com/mrcieu/ieugwasr
31. Yavorska O, Staley J. *MendelianRandomization: Mendelian Randomization Package*. 2020 [cited; Available from: https://CRAN.Rproject.org/package=MendelianRandomization
32. R Development Core Team. R: A language and environment for statistical computing. Vienna, Austria: R Foundation for Statistical Computing; 2020.
33. Hemani G, Tilling K, Davey Smith G. Orienting the causal relationship between imprecisely measured traits using GWAS summary data. *PLoS Genet* 2017; **13**: e1007081.
