# Supplementary material for: Identification of potential mediators of the relationship between body mass index and colorectal cancer: a Mendelian randomization analysis
Source: Int J Epidemiol. 2024 May 9;53(3):dyae067. doi: 10.1093/ije/dyae067 (PMC11082423; doi:10.1093/ije/dyae067)
Supplement: dyae067_Supplementary_Data [file dyae067_supplementary_data.zip › ije-2023-09-1159-File006.pdf]

**Supplementary File**

Identification of potential mediators of the relationship between body mass index and colorectal cancer: a Mendelian randomization analysis

**Contents:**

**Supplementary Figure S1.** Conceptual framework of the mediation analysis. Primarily biomarkers of cancer-related mechanisms, such as inflammation, glucose and lipid metabolism, adipokines, insulin-like growth factor 1 (IGF1)], sex-hormones, and 25-hydroxy-vitamin D (25(OH)D) were selected as mediators. Other colorectal cancer (CRC) risk factors, such as smoking, physical activity, and alcohol drinking, that are associated with body mass index (BMI) and typically act as confounders were also included in the present analysis to examine mediation versus confounding.

**Supplementary Figure S2.** Heatmap summarizing the associations between body mass index (BMI) and considered mediators, in Mendelian randomization (MR) inverse variance weighted (IVW) models. Black tiles show the nominal associations in the MR-IVW analyses ($P<0.05$), and the asterisk indicates robustness in the associations [i.e., significant associations in the MR-IVW analysis ($P<0.05$) that were qualitatively consistent in sensitivity analyses].

**Supplementary Figure S3.** Direct effects [odds ratios (OR) and 95% confidence intervals (CI)] of body mass index (BMI) on colorectal cancer (CRC) in main (denoted with round symbols) and sensitivity analyses using non overlapping single nucleotide polymorphisms (SNPs) (squares and triangles). Estimates denoted with squares were produced in Mendelian randomization (MR) analyses excluding SNPs associated with both BMI and the mediator using a *P*-value threshold of $5\times10^{-8}$, and triangles in MR analysis using a threshold of $5\times10^{-6}$, before clumping.

**Supplementary Figure S4.** Scatter plot of the genetic association estimates of body mass index (BMI)(x-axis) and insulin-like growth factor 1 (IGF1)(y-axis). Clustered heterogeneity is presented using different colours. Five distinct clusters were identified, in the association of BMI with IGF1, potentially supporting the presence of distinct pathways, inferring positive and inverse effects. The Human Genome Organisation (HUGO) Gene Nomenclature Committee (HGNC) ID of the nearest gene per single nucleotide polymorphism (SNP) was identified, and a subset of the genes (potentially supporting the presence of distinct pathways) is presented in labels.

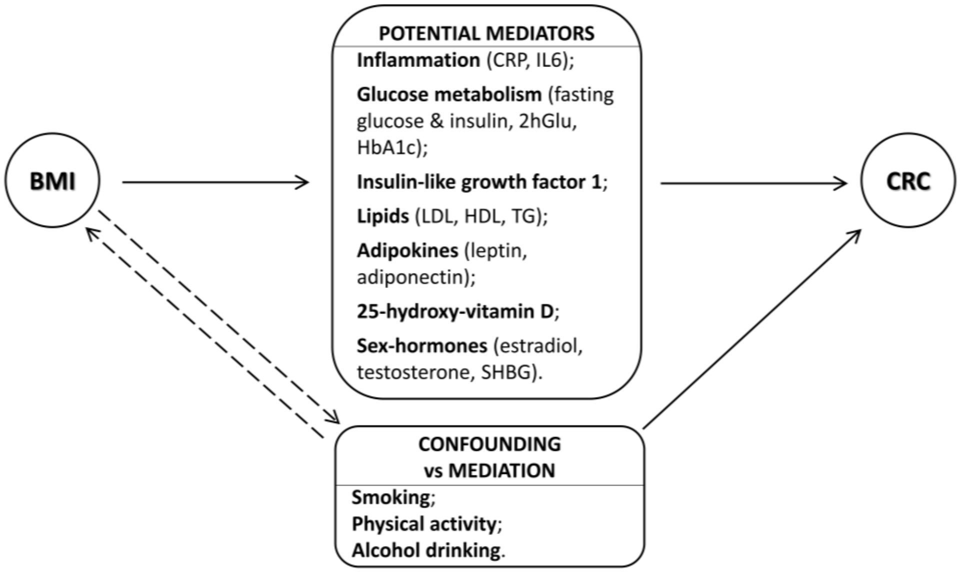


**Supplementary Figure S1.** Conceptual framework of the mediation analysis. Primarily biomarkers of cancer-related mechanisms, such as inflammation, glucose and lipid metabolism, adipokines, insulin-like growth factor 1 (IGF1)], sex-hormones, and 25-hydroxy-vitamin D (25(OH)D) were selected as mediators. Other colorectal cancer (CRC) risk factors, such as smoking, physical activity, and alcohol drinking, that are associated with body mass index (BMI) and typically act as confounders were also included in the present analysis to examine mediation versus confounding.

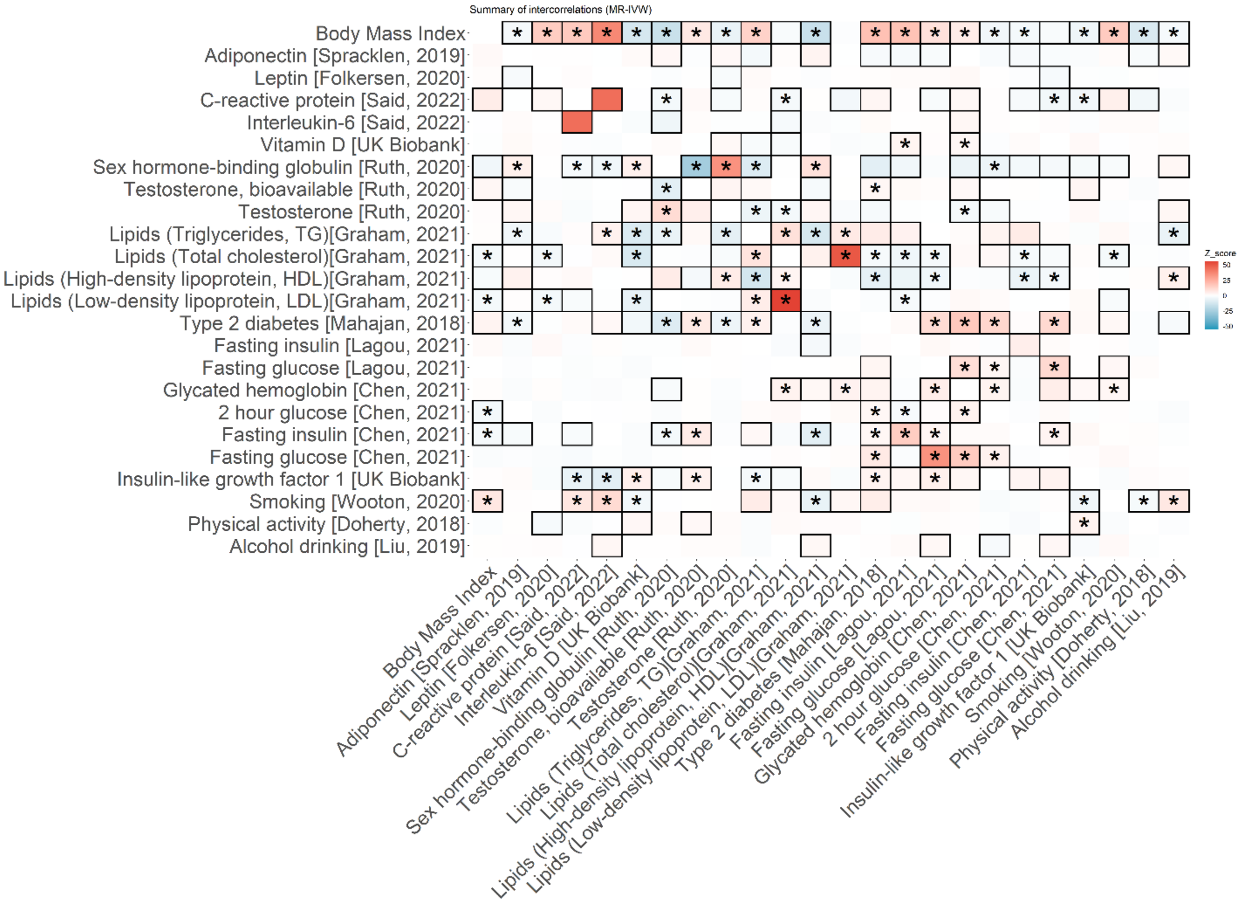


**Supplementary Figure S2.** Heatmap summarizing the associations between body mass index (BMI) and considered mediators, in Mendelian randomization (MR) inverse variance weighted (IVW) models. Black tiles show the nominal associations in the MR-IVW analyses (P<0.05), and the asterisk indicates robustness in the associations [i.e., significant associations in the MR-IVW analysis (P<0.05) that were qualitatively consistent in sensitivity analyses].

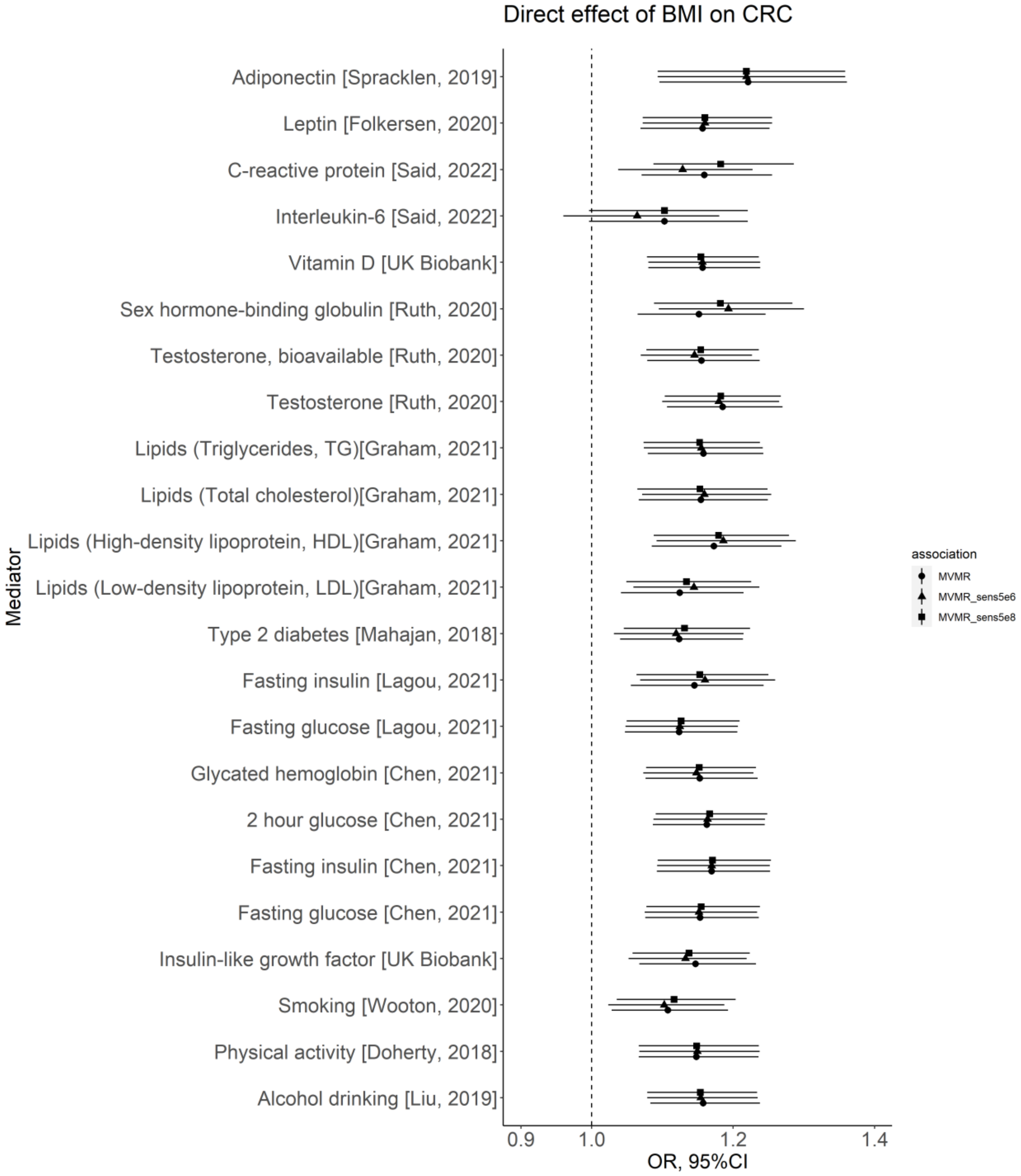


**Supplementary Figure S3.** Direct effects [odds ratios (OR) and 95% confidence intervals (CI)] of body mass index (BMI) on colorectal cancer (CRC) in main (denoted with round symbols) and sensitivity analyses using non overlapping single nucleotide polymorphisms (SNPs) (squares and triangles). Estimates denoted with squares were produced in Mendelian randomization (MR) analyses excluding SNPs associated with both BMI and the mediator using a *P*-value threshold of $5\times10^{-8}$, and triangles in MR analysis using a threshold of $5\times10^{-6}$, before clumping.

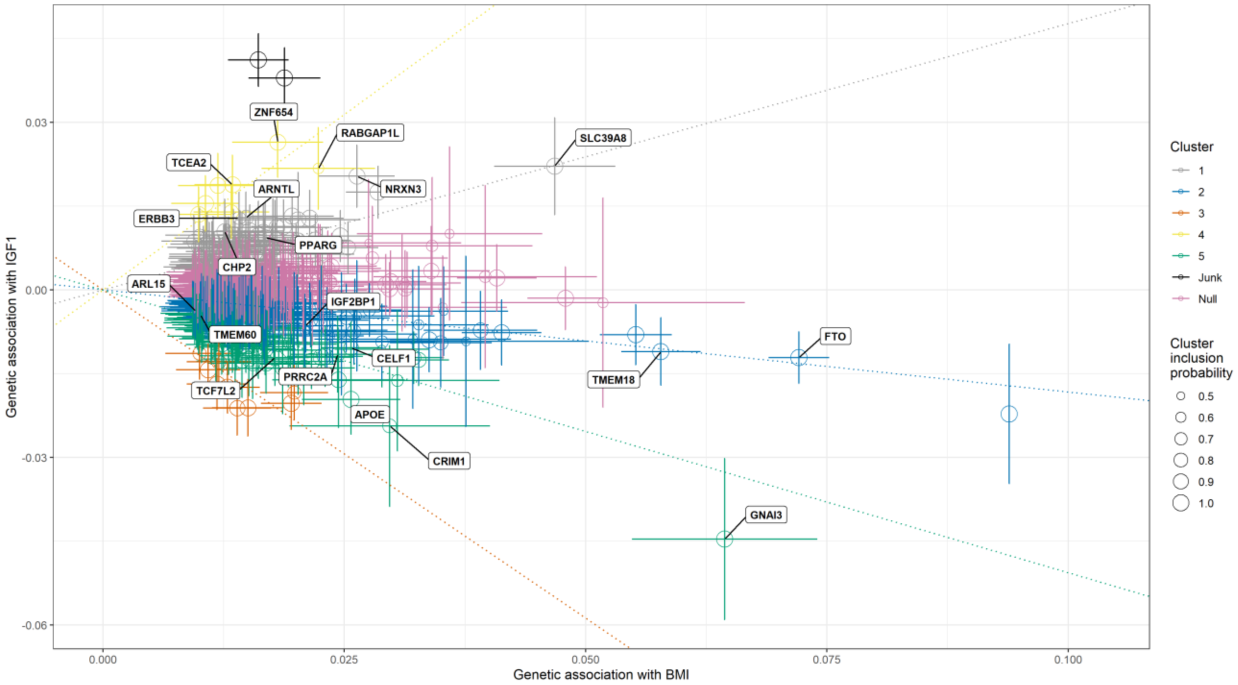


**Supplementary Figure S4.** Scatter plot of the genetic association estimates of body mass index (BMI)(x-axis) and insulin-like growth factor 1 (IGF1)(y-axis). Clustered heterogeneity is presented using different colours. Five distinct clusters were identified, in the association of BMI with IGF1, potentially supporting the presence of distinct pathways, inferring positive and inverse effects. The Human Genome Organisation (HUGO) Gene Nomenclature Committee (HGNC) ID of the nearest gene per single nucleotide polymorphism (SNP) was identified, and a subset of the genes (potentially supporting the presence of distinct pathways) is presented in labels.
